# Emerging Role of T-cell Receptor Constant β Chain-1 (TRBC1) Expression in the Flow Cytometric Diagnosis of T-cell Malignancies

**DOI:** 10.3390/ijms22041817

**Published:** 2021-02-12

**Authors:** Pedro Horna, Min Shi, Horatiu Olteanu, Ulrika Johansson

**Affiliations:** 1 Division of Hematopathology, Mayo Clinic, Rochester, MN 55905, USA; Horna.Pedro@mayo.edu (P.H.); Shi.Min@mayo.edu (M.S.); olteanu.horatiu@mayo.edu (H.O.); 2 SI-HMDS, University Hospitals Bristol and Weston NHS Foundation Trust, Bristol BS1 3NU, UK

**Keywords:** T-cell, T-cell receptor, TRBC1, clonality, diagnostics, lymphoma, leukemia, flow cytometry

## Abstract

T-cell clonality testing is integral to the diagnostic work-up of T-cell malignancies; however, current methods lack specificity and sensitivity, which can make the diagnostic process difficult. The recent discovery of a monoclonal antibody (mAb) specific for human TRBC1 will greatly improve the outlook for T-cell malignancy diagnostics. The anti-TRBC1 mAb can be used in flow cytometry immunophenotyping assays to provide a low-cost, robust, and highly specific test that detects clonality of immunophenotypically distinct T-cell populations. Recent studies demonstrate the clinical utility of this approach in several contexts; use of this antibody in appropriately designed flow cytometry panels improves detection of circulating disease in patients with cutaneous T-cell lymphoma, eliminates the need for molecular clonality testing in the context of large granular lymphocyte leukemia, and provides more conclusive results in the context of many other T-cell disorders. It is worth noting that the increased ability to detect discrete clonal T-cell populations means that identification of T-cell clones of uncertain clinical significance (T-CUS) will become more common. This review discusses this new antibody and describes how it defines clonal T-cells. We present and discuss assay design and summarize findings to date about the use of flow cytometry TRBC1 analysis in the field of diagnostics, including lymph node and fluid sample investigations. We also make suggestions about how to apply the assay results in clinical work-ups, including how to interpret and report findings of T-CUS. Finally, we highlight areas that we think will benefit from further research.

## 1. Introduction

Diagnosis of T-cell neoplasms relies on the close integration of clinical presentation and history with findings from histology and flow cytometry immunophenotyping of the relevant tissues. The presence of abnormal T-cells within the tissue may be insufficient to reach a diagnostic conclusion; therefore, molecular tests for T-cell clonality currently play an important role. These assays utilize the unique genetic fingerprint created in each developing T and B lymphocyte during the process of the T-cell receptor (TCR) and immunoglobulin (Ig) assembly [1,2]. Nonetheless, whether based on PCR or next-generation sequencing methodologies, these assays are associated with higher costs, operational complexity, and demand a high level of expertise. Moreover, these assays are prone to yield potentially false-positive results in physiologic conditions such as senescence or inflammatory states [3,4,5,6], making the diagnostic work-up of T-cell malignancies potentially difficult.

Investigation of B-cell malignancies is aided by the availability of antibodies specific for the immunoglobulin kappa and lambda light chains. Because all clonal B-cells express either kappa or lambda light chains, the ability to study the restriction of light chain expression within B-cell populations showing a disease-specific or abnormal phenotype provides additional proof of clonality. It could be argued that the use of light chain restriction analysis has, over the years, contributed to the definition of well-known B-cell malignancy immunophenotypes. Until recently, T-cell lymphoproliferations did not benefit from a readily available clonality assessment approach similar to the determination of immunoglobulin light chain restriction for B-cell lymphoproliferative disorders and required the deployment of less commonly utilized assays, such as killer immunoglobulin-like receptor (KIR) and Vβ T-cell receptor repertoire analysis [7,8,9,10,11]. However, these techniques have some limitations in being relatively expensive, labor-intensive, and requiring interpretive expertise that is not routinely available in all clinical laboratories.

The recent finding of a monoclonal antibody (mAb) specific for human TCR β chain constant region 1 (TRBC1) [12,13] opened up the possibility of a low-cost, rapid, and specific T-cell clonality test for αβTCR-positive T-cell malignancies. As discussed below, using this antibody to label T-cell populations identified according to their overall abnormal immunophenotype can yield proof of clonality in a manner similar to light chain restriction analysis. This new strategy for T-cell analysis has the potential to improve diagnostics and further our understanding of T-cell responses in health and disease.

Here, we describe how the anti-TRBC1 mAb may be included in laboratory assays, and we summarize the current knowledge-base with respect to flow cytometry-based analysis of TRCB1 during T-cell diagnostic work-up (Table 1). Additionally, we highlight areas that we consider that should benefit from further in-depth research.

## 2. Overview of T-Cell Receptor Constant β Chain Rearrangement

The TCR and Ig receptors are antigen-binding receptors fundamental to adaptive immunity because they recognize and bind a wide variety of pathogens (reviewed in [14]). This is made possible by a process called somatic recombination, which takes place during cell ontogeny and involves the rearrangement of germline genes encoding the TCR and Ig receptors [2,15]. As a consequence, each T- and B-cell expresses a unique TCR or Ig receptor. If activated due to a benign immune response, or as a consequence of malignant or autoimmune changes, the cell may undergo clonal expansion. Each daughter cell will then carry an identical TCR/Ig receptor, and this fingerprint may be used diagnostically to identify the presence of a clone.

The TCR is a transmembrane protein comprising two disulphide-linked polypeptide chains: The α and β chain or, in about 5–15% of T-cells, a γ and δ chain. Each polypeptide chain consists of a variable and a constant (C) region. The variable region contains the antigen-binding epitopes, which confer a unique receptor structure upon each individual cell (or clone). The somatic gene recombination process that shapes this part of the protein has been reviewed extensively elsewhere [16,17]. In very broad terms, sets of exons at the TCR loci (referred to as Variable (V), Diversity (D), and Joining (J) segments) are selected sequentially and then re-organized. The V and J segments are present at all TCR loci, whereas the D segments are present only at the β and δ TCR loci. This re-organization process results in so-called combinatorial diversity. Further genetic variability (so-called junctional diversity) is generated in part by the innate imprecision of the process that involves ‘cutting’ and ‘pasting’ of the V, D, and J germline rearrangements, and in part by an active, germline-independent process that adds nucleotides to the DNA joints.

Presence of T-cell clones can, therefore, be detected by PCR using primers designed to target the different V, D, and J segments, resulting in amplification of products that span these unique sequences. In theory, any TCR chain (α, β, γ or δ) may be used as a template. However, in practice, the TCRγ and TCR β chains are targeted. This is due to the way in which TCR gene rearrangement is organized during T-cell ontogeny: The TCR δ locus is rearranged first, followed by the TCRγ locus. This will result either in a functional γδTCR protein or it will be followed by rearrangement of the TCRβ locus, deletion of the TCRδ locus, and rearrangement of the TCRα locus. This, in turn, may result in a functional αβ TCR protein [15]. The TCRγ rearranged locus is retained in nearly all T-cells, including αβTCR+ T-cells, and its gene structure is simpler than that of the α or β TCR loci. Thus, the TCRγ locus is used primarily for T-cell clonality assessment [17,18,19]. Additionally, the TCRβ is used for this purpose and is favored over the α locus since the number of V and J segments in the α locus is much higher: At least 70 V and 61 J segments compared with the 64 V and 14 J segments in the β locus, respectively [20,21].

**Figure 1:**
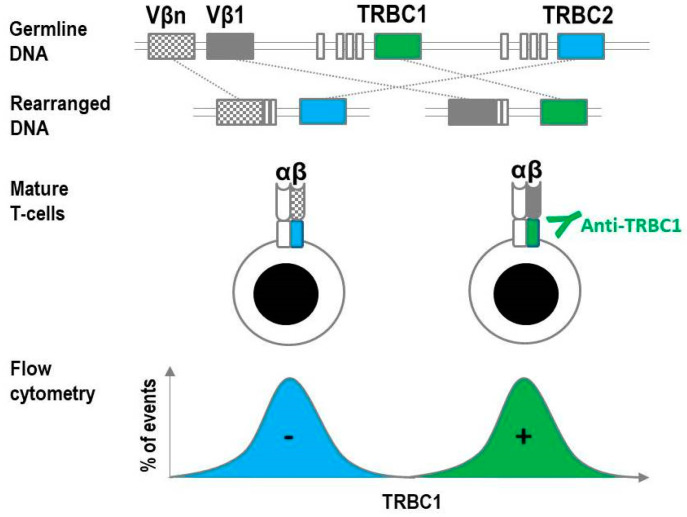
Mutually exclusive selection of two T-cell receptor (TCR) constant β chains during TCR gene rearrangement allows for the flow cytometric assessment of constant β chain monotypia as a surrogate of clonality. TCRαβ T-cell precursors in the thymus undergo rearrangement of the TCR genes, which includes the random selection of one of 2 mutually exclusive TCR constant β chains (TRBC1 and TRBC2), and one of 64 TCR variable β chains. The TCR produced from this rearrangement is expressed on the surface of all normal mature T cells. Flow cytometric analysis of normal TCRαβ T cells with a single anti-TRBC1 antibody stains only the TRBC1-positive subset, resulting in a biphasic staining pattern consistent with polytypia.

By contrast, the C (constant) region of the TCR is not part of the immediate structure that recognizes antigens. The C region loci encode the extracellular constant domain, the connecting peptide, the transmembrane domain, and the minimal intracellular domain of the TCR chains [22]. The α and γ chains each have one C region gene segment; the β and δ chains each have two segments, C1 and C2. Thus, the αβTCR on any given T-cell will have a β chain containing either C1 or C2. Since all daughter cells derived from a T-cell undergoing clonal expansion will carry identical TCRs, labeling a clonal population with an antibody specific for TCRβC1 or TCRβC2 will result in either a positive or a negative signal (Figure 1), not unlike the kappa/lambda labeling approach used for B-cell analysis. To the best of our knowledge, there is only one commercially available antibody with known specificity for a TCRβ C region (also abbreviated as TRBC) region: The TRBC1 binding monoclonal antibody JOVI-1.

## 3. Development and Characterization of the JOVI-1 (anti-TRBC1) Antibody

The only commercially available anti-human TRBC1 antibody-producing hybridoma was created nearly 30 years ago by Viney et al. [13] as they looked to produce monoclonal antibodies specific for different Vβ regions of the human TCR. The TCR variable region has poor immunogenicity, and thus, rather than using human cells or purified TCRβ chain protein as an immunogen, they used a murine cell line transfected with a TCR construct made up of a murine α chain and a human β chain that comprised of V β 3, D β 1, J β 1.2, and C β 1 as an immunogen. One of the resulting hybridomas, named JOVI-1, produced an IgG2a antibody that at the time was recognized to bind ‘a determinant’ on the majority of TCRs. However, these early studies did not yield a clear-cut picture of TRBC1 specificity. For example, flow cytometry analysis of peripheral blood or T-cell lines labeled with hybridoma culture supernatant and goat-anti-mouse FITC antibody showed that CD4^+^ T cells ranged from negative to positive, and the CD8^+^ T cells labeled biphasic; either positive or negative. Known TRBC1-positive cell lines reacted with the antibody; however, some TRBC2-expressing cell lines also showed reactivity, albeit weak. Subsequent studies using the antibody usually employed it due to its T-cell mitogenic properties or immunoprecipitation [23,24,25,26,27]. Evidence for the purified antibody’s specificity for TRBC1 was eventually published in 2017 by Maciocia et al. [12]. This group searched for TCRβ constant region specific-antibodies with the goal of creating chimeric antigen receptor T cells (CAR-T cells) specific for only TRBC1 or TRBC2-expressing T cells, a strategy that may allow T-cell directed CAR-T-cell therapy without fully depleting the patient’s T-cell compartment and adaptive immunity. The sequence and genomic organization of the two β chain C segments are known to be very similar [28], potentially making the development of highly specific mAbs more difficult. Maciocia et al., therefore, carried out extensive studies using the commercially available JOVI-1 antibody to label cell lines that expressed a variety of cloned TCR constructs. This confirmed absence of labeling of the TCR constant region 2 and moreover, that the binding of JOVI-1 to constant region 1 was not dependent on any particular TCRβ junction segment. The discriminating amino acid structural portion of the antibody’s epitope was also established [12].

## 4. Assay Design and Implementation of TRBC1 for the Detection of Clonal T cells

Appropriate assay design is of upmost importance for the utilization of TRBC1 as a T-cell clonality surrogate in clinical flow cytometry assays. Several vendors now offer the anti-TRBC1 clone JOVI.1 as a research use only (RUO) flow cytometry reagent, which can be validated by each laboratory and implemented into a routine clinical test. The detection of T-cell clones using TRBC1 is highly dependent on the evaluation of several other T-cell antigens in the same analysis tube, ideally on an 8 to 10-color flow cytometry set up, allowing for the independent assessment of immunophenotypically distinct T-cell subsets and the optimal separation of neoplastic cells from background benign, polytypic T-cells. CD3 is always needed to gate on T cells, while CD4 and CD8 are best studied in combination to separate single-positive, double-positive, and double-negative T-cell events. The expression of CD2, CD5, and CD7 is frequently abnormal and/or distinct in neoplastic T cells, facilitating their identification and gating for analysis of clonality by TRBC1. CD45 is typically added to all flow cytometry tubes, as it helps separate lymphocytes from other leucocyte subsets and CD45-negative debris, resulting in a cleaner lymphocyte gate and a better estimation of the clonal T-cell burden. Depending on the application, assessment of other antigens might be useful for the detection of specific T-cell lymphoproliferative disorders, such as CD16 and CD57 for the identification of T-cell large granular lymphocytic leukemia, CD26 for the detection of Sezary cells, and CD25 for adult T-cell leukemia/lymphoma. Coupling of the JOVI.1 antibody to a bright and highly discriminative fluorochrome is favored, as a clear separation between TRBC1-positive and TRBC1-negative events is critical for the assessment of T-cell clonality. A bright fluorochrome might also be favored for CD3 in order to more easily identify T-cell neoplasms with diminished expression of this lineage-defining antigen. CD2, CD5, and CD7 are rather brightly expressed and typically fare well with fluorochromes of intermediate brightness. CD45 is also highly expressed on lymphocytes and is, therefore, typically coupled with the less discriminatory or dimmer fluorochrome.

Some other considerations for panel design are worth noting. TCRγδ T cells are a minor small CD4-negative T-cell subset, which lacks TCRαβ expression (negative for TRBC1), and can thus be potentially misinterpreted as a clonal TRBC1-negative population when studying the CD8-positive or CD4/CD8-double-negative compartments. For many applications, routinely adding a single anti-TCRαβ or anti-TCRγδ antibody to the analysis tube provides a parameter to effectively exclude TCRγδ T cells from the clonality analysis by TRBC1. Although the CD3/TCR complex (which includes TRBC1 or TRBC2) is brightly expressed on the surface of normal mature T cells, immature T cells in the thymus have a broad spectrum of surface CD3/TCR complex expression, complicating the assessment of T-cell clonality by surface TRBC1 staining. Similar immature T cells can also be rarely encountered at low levels in lymph nodes with Castleman disease, follicular dendritic cell sarcoma, angioimmunoblastic T-cell lymphoma, and other lymph node pathologies [29]. In addition, T-cell neoplasms can occasionally be dim or negative for surface CD3/TCR. In practice, benign immature T cells can be suspected based on their characteristic immunophenotypic and maturation patterns assessed within a comprehensive T-cell panel [30], preventing them from being mistaken for clonal T cells. While a mature T-cell population with dim to negative CD3 expression might exhibit a clonal TRBC1-negative expression pattern due to lack of surface TRBC (rather than TRBC2-restriction), these immunophenotypic features are almost always indicative of a clonal T-cell lymphoproliferation or a T-cell neoplasm and should not lead to false-positive results.

The assessment of T-cell clonality using TRBC1 requires visual identification and manual gating of immunophenotypically discrete T-cell subsets and is not compatible with simplified quadrant analysis strategies. Thus, a contemporary analysis approach is needed to identify distinct T-cell populations with homogenous fluorescence properties and to independently assess the TRBC1 expression pattern on each of these populations. Competency in recognizing and gating immunophenotypically distinct subsets is, therefore, of utmost importance, as a poorly discriminative gate including both normal and malignant T cells is likely to produce a polytypic TRBC1 expression pattern. Determination of clonality on a gated and immunophenotypically distinct TCRαβ T-cell subset is based on the expression pattern of TRBC1. A largely unimodal TRBC1-negative or TRBC1-positive staining pattern is consistent with a restricted (monotypic) TCR β chain constant region expression, indicative of clonality. In addition, we have frequently encountered T-cell clones with a unimodal TRBC1-dim expression pattern, not necessarily associated with dim expression of CD3 (as would be expected in the setting of TCR downregulation) [31], and likely due to a unique TRBC1-positive TCR with diminished avidity for the JOVI.1 antibody for reasons yet unknown.

With the exception of TRBC1-dim clones, an estimate of the percentage of TRBC1-positive events is a reasonable quantitative measure to determine clonality. Novikov et al. [32] reported mean TRBC1-positive events and 95% confident intervals for normal total CD4-positive and CD8-positive T-cells in peripheral blood at 44% (36–53%) and 39% (18–61%), respectively. In patients without demonstrable T-cell neoplasia, we found a similar bimodal expression of TRBC1 on CD4-positive and CD8-positive T-cell subsets gated based on distinct immunophenotypic features, with the exception of occasional small CD8-positive T-cell subsets with a unimodal TRBC1 expression pattern [33]. Further analysis revealed that these small subsets were truly clonal based on TCR-Vβ-restriction [31] and T-cell gene rearrangement molecular studies [33], consistent with benign immunodominant clonotypes (see T-CUS section below). In our extensive experience using TRBC1 within a single-tube comprehensive T-cell panel, we have found that T-cell neoplasms virtually always have distinct immunophenotypic features that largely separate them from background benign T-cells, but that this separation based on routine gating strategies is often imperfect and results in a very skewed rather than a purely unimodal TRBC1 expression pattern. In our hands, a threshold of TRBC1-positive events less than 15% or greater than 85%, or the presence of a dominant TRBC1-dim peak, allowed for the demonstration of clonality on all TCRαβ T-cell neoplasms studied using an appropriate comprehensive panel, and confirmed polyclonality on all benign T-cell subsets with the exception of occasional small and benign immunoclones.

In general, a comprehensive single T-cell tube with TRBC1, CD45, TCRγδ and all core T-cell antigens (CD2, CD3, CD4, CD5, CD7, and CD8) might be a good initial approach to evaluate for clonal T cells compatible with a T-cell malignancy. This upfront assessment of T-cell clonality within a single T-cell immunophenotyping tube differs from conventional practice, where a separate T-cell clonality assay (typically PCR using BIOMED-2 primers, and less commonly TCR Vβ analysis by flow cytometry) is reflexed in selected cases only. Indeed, the European Consortium of flow cytometry laboratories EuroFlow currently recommends an initial single-tube assessment of B and T lymphocyte subsets, followed by a reflex T-cell clonality testing using an 8-tube TCR Vβ analysis on selected cases, and a third step to characterize the detected clonal T-cell subset using six additional analysis tubes [34]. Except for large reference centers, most flow cytometry laboratories have found that TCR Vβ flow cytometry is too labor-intensive for routine implementation, and the long turn-around-time of T-cell clonality by PCR precludes rapid integration within the flow cytometry workflow. Upfront T-cell clonality using a single anti-TRBC1 antibody largely simplifies this process and is likely to be strongly considered by the EuroFlow consortium and flow cytometry laboratories of all sizes. Integration of T-cell clonality assessment by TRBC1 into specialized analysis tubes for the detection of specific T-cell neoplasias, such as cutaneous T-cell lymphomas and cytotoxic T-cell neoplasms, might also be useful depending on the practice scope. Interpretation of the findings should take into account the limitations of T-cell clonality assessment by TRBC1, including:

(1) Inability to detect clonal gamma/delta T-cells;
(2) Limited ability to detect clonal T cells that are not immunophenotypically distinct with the combination of antigens studied;
(3) Common detection of small T-cell clones of uncertain significance in patients without T-cell neoplasia (see below).

## 5. T-cell Clones of Uncertain Significance (T-CUS) Detected by TRBC1 Staining

The normal immune function of T-cells requires the clonal expansion and persistence of small T-cell subsets sharing the same TCR in order to mount a targeted response against a recognized epitope, escalate such a response, and develop immune memory [35]. Highly sensitive T-cell clonality assays have demonstrated that dominant T immunoclones generated during immune responses are ubiquitous in health and disease [36,37,38,39,40,41,42]. Such clones are much more predominant in the CD8-positive compared to the CD4-positive T-cell compartment, and they are frequently associated with responses to common viral infections and neoplasms [36,37,38,39,40,42]. Routine clinical assays of T-cell clonality are typically not sensitive enough to detect dominant immunoclones in young, healthy individuals but can occasionally produce equivocal or positive T-cell clonality results in patients with reactive inflammatory conditions and with aging [43].

**Figure 2:**
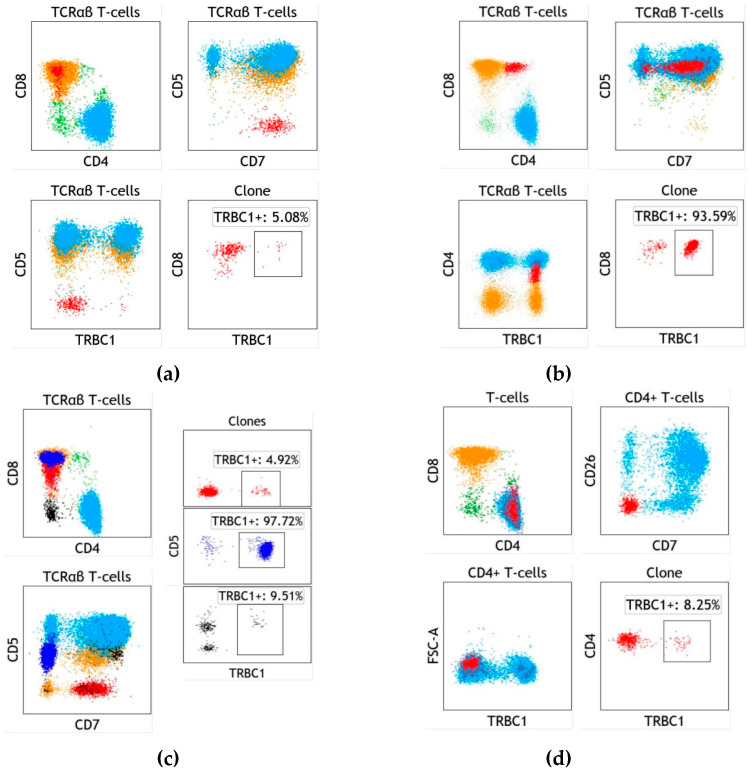
T-cell clones of uncertain significance (T-CUS), detected in patients with no clinical evidence of T-cell malignancy. (**a**) Typical CD8-positive T-CUS (red) detected in a bone marrow aspirate from a patient with splenic marginal zone lymphoma. The clone is relatively small in size, TRBC1-negative, and easily identifiable on a CD5 vs. CD7 dot plot. (**b**) CD4/CD8 double-positive T-CUS (red) in a peripheral blood specimen from a patient status post-renal transplant. This variant frequently exhibits dim CD4 or CD8 expression. The clonal T cells are TRBC1-positive. (**c**) Multiple T-CUS identified in a peripheral blood specimen from a patient with inclusion body myositis and normal blood counts. Two dominant CD8-positive clones (red and blue) are identified on a CD5 vs. CD7 dot plot, one positive and another negative for TRBC1. A third small CD4/CD8 double negative T-CUS lacking TRBC1 expression (black) is also detected. (**d**) A CD4-positive T-CUS (red) is detected in a blood specimen from a patient with metastatic squamous cell carcinoma and chronic lymphocytic leukemia. These rare and small clonal subsets are frequently negative for CD7 and CD26 and often cluster within the higher normal range for light scatter. In addition, shown are polytypic CD4-positive (cyan) and CD8-positive (orange) T cells and other non-gated events (green). T cells were gated based on CD3 expression, and TCRαβ T cells were gated by the exclusion of TCRγδ positivity. FSC-A: Forward light scatter amplitude.

The integration of clonality assessment by TRBC1 into routine clinical practice has resulted in the frequent identification of small T-cell clones of uncertain significance (T-CUS) in patients with no clinical evidence of T-cell lymphoma [31], most likely representing dominant T immunoclones (Figure 2). As predicted from prior reports, most T-CUS detected by TRBC1 staining has an immunophenotype reminiscent of CD8-positive T-cell large granular lymphocytes, with a clone size that only occasionally overlaps with that seen in T-cell large granular lymphocytic leukemia (T-LGLL) [44]. Interestingly, we found no association between the presence of T-CUS and clinical features typically encountered in T-LGLL (namely cytopenias, autoimmune diseases, or decreased numbers of NK cells). Despite the close immunophenotypic similarities between T-CUS and T-LGLL, these two clonal proliferations are likely representative of physiologically different processes, one resulting from a T-cell immune response (T-CUS) and the other secondary to the unhindered expansion of malignant T-cells (T-LGLL). Follow up studies of patients with T-CUS and genomic characterization of sorted clonal T-cells are needed to further explore the potential relationship between T-CUS and T-LGLL.

While T-CUS is by far most commonly encountered in the CD8-positive/CD4-negative T-cell compartment, other less common immunophenotypic variants are worth noting. A CD4/CD8 double-positive T-CUS has been described in the setting of T-cell responses to CMV [45,46], often exhibiting dim expression of CD4 or CD8 (Figure 2b), and most of the time not raising concern for T-LGLL, which generally does not show these distinct immunophenotypic features. A small CD4-positive T-CUS can also be rarely encountered (<150 cells/μL)(Figure 2d), particularly when studying CD4-positive subsets on a CD7 vs. CD26 dot plot for the detection of Sezary cells [47]. The absence of tumor-specific immunophenotypic abnormalities, presence of light scatter properties in the upper end of the normal spectrum, and expression of CD57 on a few cases studied (personal experience) raise the possibility of a small reactive CD4-positive large granular lymphocyte subset undergoing similar physiologic clonal expansions as CD8-positive T-cells. However, the biological or clinical significance of CD4-positive T-CUS has not yet been adequately studied.

As T-cell clonality assessment by TRBC1 is implemented in more diagnostic flow cytometry laboratories, we anticipate that the concept of T-CUS will be utilized more routinely in order to best interpret the presence and immunophenotype of small T-cell clones. Given the high prevalence of CD8^+^/CD4^−^ T-CUS, an arbitrary threshold of 15% of all lymphocytes for a clonal CD8-positive T-cell population lacking malignancy-specific immunophenotypic abnormalities (e.g., conspicuously dim expression of CD2, CD3, or CD45; or conspicuously increased light scatter) might be a good practice guide to determine when to raise concern for a CD8-positive T-cell lymphoproliferative disorder [31]. In these common variants of T-CUS, loss of CD5 and/or CD7 is frequently encountered and should not by itself raise concern for neoplasia. A similar threshold could be applied for CD4^+^/CD8^dim^ and CD4^dim^/CD8^+^ small T-cell subsets, which are, in general, most consistent with T-CUS. CD4^+^/CD8^−^ T-CUS is much more infrequent and might be best reported at any detectable size, especially if the clinical context is not known by the laboratory. Malignancy-specific immunophenotypic abnormalities in CD4^+^/CD8^−^ T-cells (e.g., conspicuously dim expression of CD2, CD3, CD4, CD5, or CD45; or conspicuously increased light scatter) should further prompt strong consideration of a T-cell neoplasm in the appropriate clinical setting.

For the most part, T-cell lymphoproliferative disorders typically show distinct tumor-specific immunophenotypic abnormalities that allow for the detection of low-level and minimal residual disease in the appropriate clinical setting and supported by clonality assessment using TRBC1 staining. However, for some diseases like T-LGLL, the extensive immunophenotypic overlap with T-CUS precludes the confident detection of low-level disease in a significant subset of cases. This is further complicated by frequent immunophenotypic shifts encountered in clinical practice on follow-up specimens. Thus, the ability to distinguish T-CUS from low-level/minimal residual T-cell neoplasia varies for each specific disease category and depends on the presence and persistence of distinctive tumor-specific immunophenotypic features.

## 6. Specific Case of T-cell Large Granular Lymphocytic Leukemia

T-cell large granular lymphocytic leukemia (T-LGLL) is a chronic lymphoproliferative disorder characterized by the clonal expansion of cytotoxic T-cell large granular lymphocytes. It may emerge from an uncontrolled clonal outgrowth of large granular T lymphocytes stimulated by self, viral, or tumor antigens. T-LGLL accounts for approximately 2–5% of the chronic lymphoproliferative disorders, and it usually affects older individuals with a medium age of 60 years-old at diagnosis [48,49]. Most T-LGLL patients present with cytopenia(s), and commonly in association with autoimmune diseases and other hematopoietic malignancies [48]. Approximately two-thirds of patients eventually develop severe symptoms that require treatment. T-LGLL typically involves peripheral blood and bone marrow; it may also infiltrate the liver and spleen, causing hepatosplenomegaly.

Diagnosing T-LGLL requires a collective assessment of morphology, immunophenotypic aberrancy, T-cell clonality, and bone marrow biopsy, in conjunction with clinical presentation. T-LGLL has small nuclei and ample cytoplasm containing prominent azurophilic granules with minimal cytological atypia. Although increased circulating large granular lymphocytes are a typical feature of T-LGLL [50], a T-cell large granular lymphocyte (T-LGL) count of 2 × 10^9^/L is no longer required to diagnose T-LGLL because not uncommonly, T-LGLL cases have a T-LGL count lower than 1 × 10^9^/L [51]. The tumor cells characteristically show co-expression of one or more natural killer (NK)-cell-associated antigens (CD16, CD56, or CD57) and decreased CD2, CD5, or CD7 expression. Of note, the morphologic and immunophenotypic features of T-LGLL are not specific and have significant overlap with those of reactive T-LGL expansions. Approximately 80% of T-LGLL cases reveal intrasinusoidal cytotoxic T-cell infiltrates in the bone marrow, which has not been observed in cytopenic patients with a reactive increase in blood T-LGLs [52]. Currently, T-cell clonality is established by positive TCR gene rearrangement (TCGR) using the standard BIOMED-2 polymerase chain reaction (PCR) assay, TCR Vβ immunophenotyping by flow cytometry, or killer-cell immunoglobulin-like receptor (KIR) flow cytometry study [9]. However, TCGR has a long turnaround time and cannot correlate clonality with immunophenotypic aberrancy; TCR Vβ flow cytometry is labor-intensive and is a low-sensitive assay covering only 70% Vβ genes; and KIR expression by flow cytometry is not widely performed.

**Figure 3:**
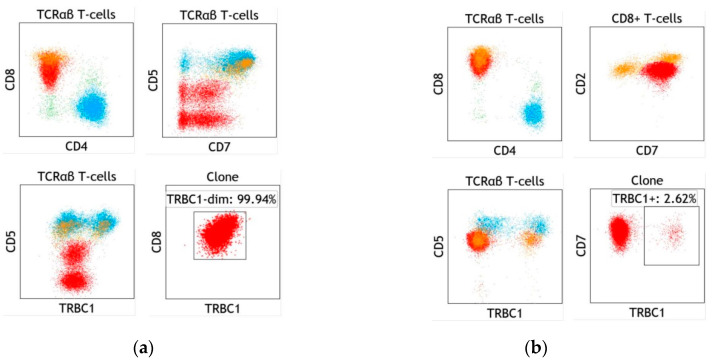
Examples of T-cell large granular lymphocytic leukemia (T-LGLL), showing an expanded CD8-positive T-cell subset with a clonal TRBC1 staining pattern. (**a**) Classic example of T-LGLL in a bone marrow aspirate, characterized by a dominant CD8-positive T-cell population (red) with an aberrant dim expression of CD8 and diminished to negative CD5 and CD7 expression. Monophasic TRBC1-dim expression on both the CD5-dim and CD5-negative subsets is consistent with a single large clonal population. (**b**) A case of T-LGLL in a bone marrow aspirate, lacking conspicuous immunophenotypic abnormalities. The flow cytometry findings are characterized by a dominant CD8-positive subset (red) with discrete clustering on a CD2 vs. CD7 dot plot and a clonal TRBC1-negative expression pattern. In addition, shown are polytypic CD4-positive (cyan) and CD8-positive (orange) T cells and other non-gated events (green). TCRαβ T cells were gated by the exclusion of TCRγδ positivity.

With the recently reported flow cytometric strategy to assess T-cell clonality using a TRBC1 antibody against one of two mutually exclusive TRBC regions [32,33], T-LGLL has been recognized as an excellent example to use this strategy for T-cell clonality because 1) almost all T-LGLLs show surface CD3 expression; 2) 95% of T-LGLLs show TCR αβ type, although intermittent TCR γδ type [53] and rare mixed-phenotype occur [54]. Incorporating TRBC1 into routine flow cytometric panels makes clonal T-cell identification from the reactive background T-cells possible (Figure 3). In our experience, all T-LGLL cases revealed monotypic TRBC1 expression, concordant with the clonal/equivocal TCR gene rearrangement results [44]. In contrast, only one-fourth of T-LGLL cases showed clonality based on the expression of restricted KIRs. This finding indicates TRBC1 assessment by flow cytometry was comparable to TCGR molecular study and superior to KIR flow cytometric analysis in demonstrating T-cell clonality in T-LGLL. Given the 100% sensitivity of TRBC1 assessment by flow cytometry in identifying T-cell clonality, we could consider using this simple, rapid, and reliable assay to replace other methodologies for T-cell clonality assessment in patients suspicious for T-LGLL.

However, immunophenotypically distinct T-cells that express NK-cell associated markers and monotypic TRBC1 do not equate to T-LGLL cells because they may arise from benign clonal T-cell expansions in patients without demonstrable T-cell neoplasia (T-CUS). As mentioned above, T-CUS is morphologically and immunophenotypically indistinguishable from T-LGLL. TRBC1 clonality assessment, together with immunophenotypic aberrancy, allows for accurate quantitation of clonal T-cells. Using this methodology, we found that T-CUS has a much lower clonal T-cell count than T-LGLL, although overlap in clonal size has been noticed [31]. The median clonal T-cell count in T-LGLL patients was 2376 cells/µL (range 198.8–29,905.9 cells/µL), which comprised of 66.0% (range 14.5–97%) of total lymphocytes [31]. In contrast, a median clone size in T-CUS was at 38 cells/uL (range 2.5–829 cells/uL), consisting of 3.3% (range 0.2–66%) of total lymphocytes. To avoid overcalling the highly prevalent T-CUS as T-LGLL, we have empirically used the clonal T-cell percentage ≥15% of total lymphocytes or a clonal T-cell count ≥500 cells/μL as cutoffs to rule in the potential diagnosis of T-LGLL. For patients who have a lower number of clonal T-cells, a bone marrow biopsy could be performed to further evaluate the significance of the small clone, if clinically indicated. These cutoffs should be only applied for the diagnostic, not follow-up specimens. Occasionally, a reactive process could have a clonal T-cell expansion of more than 15% of total lymphocytes or over 500 cells/μL. However, this large clonal expansion is usually a temporary reaction to acute infection/inflammation; over time, the clone will diminish or disappear.

**Figure 4:**
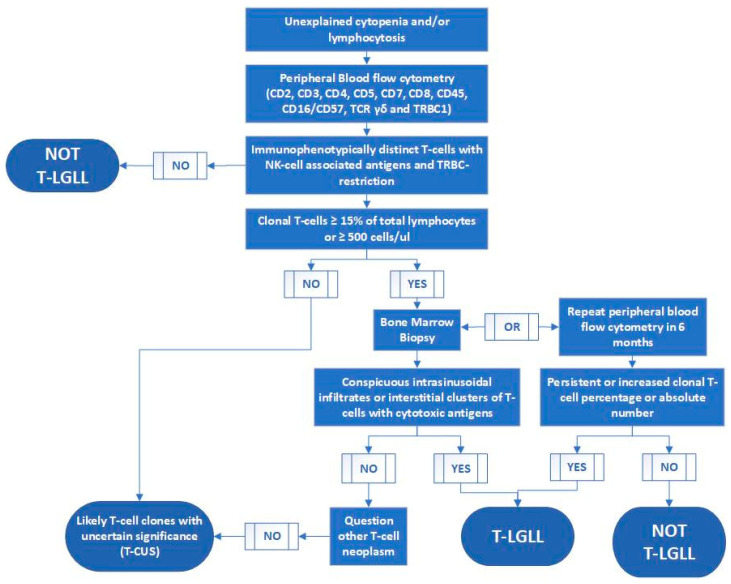
Laboratory work-up of patients with clinical suspicion for T-cell large granular lymphocytic leukemia (T-LGLL).

Given the significant overlapping features between T-LGLL cells and their reactive counterparts, we propose to reestablish/refine the diagnostic criteria of T-LGLL by incorporating TRBC1 into the routine flow cytometry assay. In this T-LGLL work-up, we propose to use TRBC1 clonality assessment to replace the traditional TCGR, TCR Vβ immunophenotyping, and KIR analysis, thus facilitating the diagnosis of T-LGLL (Figure 4). When T-LGLL is suspected in a patient with cytopenia(s) and/or lymphocytosis, peripheral blood flow cytometry should be performed using a comprehensive antibody panel against T-cell associated antigens (CD2, CD3, CD4, CD5, CD7, CD8, CD45, TRBC1, TCRγδ) and NK cell-associated antigens (CD16 and CD57). Lack of a TRBC1-restricted T-cell population essentially rules out the diagnosis of T-LGLL. The presence of a small TRBC1-restricted αβ T-cell clone, representing less than 15% of total lymphocytes and <500 cells/μL, renders T-LGLL unlikely (probable T-CUS). T-LGLL is suspected when a large TRBC1-restricted αβ T-cell population with NK-cell associated antigens is detected, that is >15% of total lymphocytes or ≥500 cells/μL. Further work-up may be required to confirm the diagnosis of T-LGLL. A bone marrow biopsy is recommended if other myeloid and/or lymphoid malignancies are in the differential diagnoses. A characteristic intrasinusoidal distribution or interstitial clusters of cytotoxic T-cells in the bone marrow biopsy renders a diagnosis of T-LGLL. If bone marrow biopsy is not desired, establishing a temporal persistence of a T-cell clone is necessary to rule out a potentially transient expansion of a physiologic T-cell clone in response to infections. We recommend repeating peripheral blood flow cytometry in more than six months. If the size of the T-cell clone remains stable or increased, T-LGLL is confirmed. Sometimes, a bone marrow biopsy to establish the characteristic bone marrow finding is preferred to avoid waiting for at least six months to repeat peripheral blood flow cytometry. STAT3 and STAT5b mutations have been identified in T-LGLL and may correlate with disease features [55,56]. However, they are not necessary for T-LGLL diagnosis because:

(1) STAT3 and STAT5b mutations have been detected in approximately 50% and 2% of T-LGLL patients, respectively, indicating low sensitivity;
(2) STAT3 or STAT5b mutations have been found in patients with Felty syndrome, aplastic anemia, myelodysplastic syndrome, and other T/NK-cell neoplasms, suggestive of low specificity [57,58,59,60,61];
(3) Molecular study for STAT3 or STAT5b mutations is not broadly available and typically has a long turnaround time.

## 7. Specific Case of Blood Involvement by Cutaneous T-cell Lymphoma

Mycosis fungoides and Sezary syndrome are two distinct but intimately related T-cell lymphoproliferative disorders involving the skin, commonly referred together as cutaneous T-cell lymphomas (CTCLs) [62,63]. Mycosis fungoides typically presents with slowly progressing patch and plaque lesions, while Sezary syndrome is characterized by extensive skin involvement at presentation in the form of erythroderma, in addition to significant blood and lymph node involvement. Disease staging and assessment of therapy response in CTCL require a quantitative assessment of peripheral blood involvement (blood rating) in absolute number of neoplastic cells (Sezary cells) per microliter (B0: <250, B1: ≥250 and <1000, and B2: ≥1000 Sezary cells/μL) [64,65], with important prognostic and therapeutic implications [66,67]. Nowadays, flow cytometry is the method of choice to estimate the number of Sezary cells in peripheral blood, largely replacing the previously utilized, subjective, and time-consuming microscopic quantitation of atypical lymphocytes on a Wright-stained peripheral blood smear. While some groups have previously advocated for a simplified flow cytometry approach based on the quantification of CD4^+^ T cells lacking CD7 and/or CD26 expression (most common Sezary cell immunophenotype) [65,68], others have found this approach to be suboptimal, as these immunophenotypic features are not specific for neoplasia and consistently include benign T-cell subsets, which are commonly expanded in reactive conditions [69,70,71]. A recent consensus statement by flow cytometry experts recommended the additional assessment of abnormalities that are more specific for neoplasia, such as dim expression of CD3, CD4, and CD45, or increased light scatter properties [72,73,74]. However, these neoplasia-associated immunophenotypic abnormalities are inconsistently present, typically subtle, and often times equivocal or absent [69,70,72,73]. Few reference centers have opted to query for T-cell clonality using a set of 24 TCR-Vβ class-specific antibodies by flow cytometry [7,75], an approach that is time-consuming, of limited sensitivity, and not accessible to most diagnostic laboratories. Finally, T-cell receptor gene rearrangement studies performed in a separate PCR assay can often be helpful [3,4] but are of limited specificity for T-cell neoplasia [5] and do not provide immunophenotypic or quantitative information about the clone detected. 

**Figure 5:**
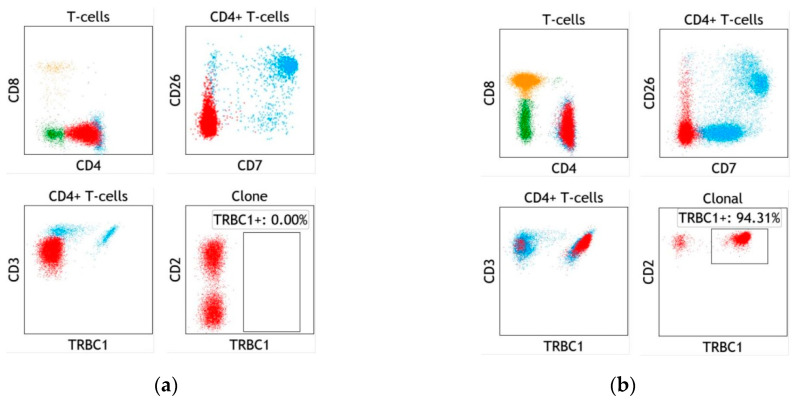
Examples of peripheral blood involvement by cutaneous T-cell lymphoma, showing TRBC1-restricted CD4-positive T cells consistent with Sezary cells. (**a**) A blood sample from a patient with advanced mycosis fungoides shows typical CD4-positive Sezary cells (red) with aberrant diminished expression of CD3 and CD4, and negative for CD7 and CD26, accounting for 991 cells/μL. Two clusters are identified based on CD2 expression, both of which likely correspond to the same TRBC1-negative T-cell clone. (**b**) A case of Sezary cells with no detectable tumor-specific immunophenotypic abnormalities in a patient with mycosis fungoides. The conventional immunophenotypic analysis shows only a relative increase in CD4-positive T cells lacking CD7 and/or CD26, a feature also observed in some reactive settings. Analysis of TRBC1 expression on CD4-positive T-cells shows T-cell clonality in the CD7/CD26 double-negative subset (red), but not in the expanded CD7-dim/CD26-negative population, consistent with a Sezary cell count of 585 cells/μL. In addition, shown are background polytypic CD4-positive (cyan) and CD8-positive (orange) T cells and other non-gated events (green). T cells were gated based on CD3 expression, and absolute Sezary cell counts were estimated by correlation with the absolute lymphocyte count obtained separately on a hematology analyzer.

A single anti-TRBC1 antibody added to a comprehensive Sezary cell flow cytometry panel provides an elegant, low-cost, and biologically sound approach to confidently and reproducibly quantify Sezary cells. In particular, reactive CD4-positive T-cell subsets lacking CD7 and/or CD26, or exhibiting unusual immunophenotypic properties, can be easily distinguished from Sezary cells based on the absence or presence of a clonal TRBC1 staining pattern, respectively. Conversely, Sezary cells lacking tumor-specific immunophenotypic abnormalities can be rapidly identified as clonal, based on TRBC restriction. In addition, complex cases comprised of more than one immunophenotypically distinct Sezary cell subset can be easily identified as a single neoplastic population, based on an identical and clonal TRBC1 expression pattern, resulting in a more confident and accurate quantitation of neoplastic cells for staging (Figure 5). This approach is accessible to all laboratories that perform comprehensive leukemia/lymphoma immunophenotyping and essentially eliminate the need for a separate flow cytometric or molecular T-cell clonality assay.

In our practice, we have demonstrated the superiority of TRBC1 evaluated within a Sezary cell panel [47], compared to a simplified strategy of quantifying CD4-positive T cells lacking CD7 or CD26 expression recently endorsed by the European Organization of Research and Treatment of Cancer (EORTC) [64], and to comprehensive immunophenotyping alone (without TRBC1) [76]. Importantly, the EORTC approach consistently produced false Sezary cell counts in 88 patients with no demonstrable T-cell neoplasia, with a limit of blank (LOB, 95th percentile) of 445 Sezary cells/μL, which is well above the 250 cells/μL threshold for B1 blood rating. Indeed, false B1 blood ratings were produced in 16 (18%) of these patients without T-cell lymphoma. In sharp contrast, the TRBC1 method resulted in a LOB of only 46 cells/μL (due to rare small CD4-positive T-CUS) and no false B1 blood ratings. When studying 111 blood samples from patients with CTCL, the absolute Sezary cell counts produced by the EORTC and TRBC1 methods were almost identical above 500 Sezary cells/μL (upper B1, and B2 ratings). Below this threshold and as expected from studying patients with no T-cell lymphoma, the EORTC method consistently produced higher Sezary cell counts, even in the absence of clonal CD4-positive T cells detectable by TRBC1. Comprehensive immunophenotyping alone (no TRBC1 analysis) did not show CD4-positive T-cells with tumor-specific immunophenotypic abnormalities in any of the patients without T-cell lymphoma, or in CTCL patients with no clonal CD4-positive T-cells detected by TRBC1 staining. However, this approach failed to demonstrate tumor-specific immunophenotypic abnormalities in 13 of 56 (23%) clonal Sezary cell populations identified by TRBC1 staining, including 2 B1-rated and 4 B2-rated clones [47]. Three important conclusions can be drawn from these results:

(1) Immunophenotypic analysis with assessment of clonality by TRBC1 has a far superior test performance for the detection of Sezary cells within the B0–B1 blood rating range, compared to the EORTC method;
(2) The TRBC1 strategy can safely replace the EORTC method without significant impact on B2 blood rating;
(3) Clonality assessment by TRBC1 effectively overcomes the limitations of comprehensive immunophenotypic analysis to identify Sezary cells lacking tumor-specific immunophenotypic abnormalities confidently.

The unprecedented analytical sensitivity of TRBC1 to confidently detect small clonal T-cell populations provides an opportunity to accurately study the prognostic significance of blood involvement by CTCL at levels below the threshold for B2 blood rating. Indeed, the seminal reports that defined the clinical relevance of blood involvement in CTCL relied either on microscopic evaluations or simplified flow cytometry strategies with limited analytical sensitivity [65,66,67]. While the current CTCL staging system does not grant much weight on B1 blood involvement compared to B0, at least one large study has shown the independent and adverse prognostic relevance of a positive T-cell gene rearrangement result by molecular methods [66], suggesting that low-level peripheral blood involvement might be more prognostically relevant than previously thought. In our implementation of TRBC1 staining, we have been able to detect and quantify minute CD4-positive T-cell clones down to approximately 10 cells/μL, when acquiring 200,000 total events. Higher event acquisition is common in routine practice, and a test design for minimal residual disease (MRD) analysis in the setting of CTCL is feasible. One caveat for MRD testing is the detection of uncommon and very small CD4-positive T-CUS (usually below 50 cells/μL) [47]. Luckily, most Sezary cell populations do exhibit immunophenotypic properties distinguishable from reactive subsets and can be confidently identified as such, even at very low numbers.

## 8. Applications of TRBC1 Assessment for Tissue Analysis and Low-Cellularity Specimens

Several categories of T-cell neoplasms tend to present predominantly in extramedullary sites, including tissue and body fluids. Entities such as peripheral T-cell lymphoma, not otherwise specified (PTCL, NOS), mycosis fungoides/Sezary syndrome (MF/SS), extranodal NK/T-cell lymphoma, nasal type (ENKTCL), angioimmunoblastic T-cell lymphoma (AITL), T-cell lymphoblastic lymphoma (T-ALL), or anaplastic large cell lymphoma (ALCL), are often diagnosed primarily in lymph nodes, skin or other extramedullary sites. Body fluids may be the primary or (more often) the secondary site of involvement by some of these malignancies.

The diagnosis of T-cell lymphomas involving tissue biopsies has relied traditionally on a combination of histomorphologic features and possibly supplemented by immunohistochemistry, molecular, and/or genetic analysis. Although multiple studies have documented the utility of flow cytometric immunophenotyping in identifying the neoplastic nature of a T-cell infiltrate, with high sensitivity and specificity, its application in the diagnosis of T-cell neoplasms has been variable in practice [77,78,79,80]. The reason for that resided in the absence of an easy means of establishing T-cell clonality by flow cytometry, which led to it being viewed as a less important technique in the ancillary testing armamentarium.

We have recently expanded on our flow cytometric strategy to assess T-cell clonality using a single TRBC1 antibody (JOVI-1), to include tissue and body fluid specimens, in addition to peripheral blood and bone marrow samples [81]. Our evaluation of 143 tissue and body fluid specimens, comprising both patients with a definitive diagnosis of a T-cell neoplasm and patients with no T-cell malignancy, included a broad array of specimen types from multiple anatomic sites (lymph nodes, spleen, lung/mediastinum, tonsil, gastrointestinal/liver, nasopharynx, and soft tissue), as well as pleural, peritoneal, and cerebrospinal fluids. The neoplastic diagnoses were also diverse and consisted of PTCL, NOS; AITL, MF/SS, PTCL, T-follicular helper type (PTCL-TFH), T-cell prolymphocytic leukemia, ALCL, ENKTCL, and hepatosplenic T-cell lymphoma. All cases of mature T-cell lymphomas with documented neoplastic T-cell populations showed restricted (monotypic) TRBC1 expression, a sensitivity of 100%. We also established the expression patterns of TRBC1 in normal T-cell subsets from tissue and body fluid cases without a T-cell neoplasm, and we demonstrated that T-cell malignancies show a narrow and distinctly restricted (positive or negative) TRBC1 expression [81]. T-cell receptor gene rearrangement (TCR-PCR) studies were available in 38/46 (82.6%) of tissues or body fluids diagnosed with a T-cell neoplasm and in 8/97 (8.3%) of specimens without a T-cell malignancy. There was 100% concordance between a clonal TCR-PCR result and restricted TRBC1 expression in cases with a neoplastic T-cell clone. None of the eight specimens without T-cell malignancy showed a positive TCR-PCR. In addition, eight samples without T-cell malignancy and with a negative TCR-PCR also showed polytypic TRBC1 expression by flow cytometry. This comparison with clonality assessment by molecular analysis indicated that the flow cytometric TRBC1 assay had comparable sensitivity and specificity while being faster and less costly. Furthermore, TCR-PCR testing provides only a global assessment of the submitted sample and a binary output (clonal vs. non-clonal population present), whereas TRBC1 testing yields specific clonality information on distinctly aberrant T-cell populations, as defined by immunophenotypic analysis. In addition, our cohort was analyzed with our routine diagnostic T-cell panel that additionally included the anti-TRBC1 mAb, demonstrating that this robust and accurate approach may be easily implemented in the clinical workflow in a wide variety of laboratories. As such, TRBC1 assessment by flow cytometry may change the way other ancillary testing is being used for T-cell clonality assessment. We anticipate that the presence of a clonal (monotypic) TRBC1 flow cytometry result will obviate the need to perform a more costly and time-consuming test, at least in a subset of cases.

While a diagnosis of T-cell lymphoma may be rendered in a sufficient number of cases based on histologic identification of an overtly malignant infiltrate alone, the utility of our proposed approach was particularly impactful in the subset of tumors that showed significant morphologic overlap with other reactive or neoplastic processes. In those cases, the inclusion of TRBC1 assessment by flow cytometry may allow for rapid confirmation of the neoplastic cell lineage. We have encountered in our practice cases of borderline atypical lymph node infiltrates that required multiple biopsies over the span of several months in order to eventually substantiate the possibility of T-cell lymphoma based on immunophenotypic analysis that demonstrated restricted TRBC1 expression. Our cohort consisted primarily of excisional or incisional lymph node biopsies but also of fine needle aspirate and core biopsy specimens. The latter category may include a higher proportion of paucicellular cases, and the increased sensitivity and specificity conferred by TRBC1 assessment is particularly useful in the evaluation and classification of T-cell neoplasms in those circumstances.

In summary, our clinical experience supports the usefulness of TRBC1 assessment in the diagnosis of T-cell neoplasia in tissue and body fluid specimens, including low-cellularity samples. We found this approach to be very sensitive, demonstrating restricted TRBC1 expression in a large cohort of cases covering the spectrum of mature, surface CD3-positive T-cell malignancies. In addition, when interpreted in the context of a detailed assessment of normal and reactive T-cell compartments, as part of an analytic strategy that emphasizes accurate gating of immunophenotypically distinct T-cell subsets, it is also highly specific. 

## 9. Future Developments

As the utilization of TRBC1 staining to detect T-cell clonality continues to expand to many flow cytometry laboratories, more evidence is emerging regarding its high diagnostic and analytical performance, low cost, and applicability to various T-cell neoplasms and specimen types. The JOVI.1 antibody provides a clear separation between TRBC1-positive and TRBC1-negative (TRBC2-positive) events, as it is by itself sufficient to assess for TRBC-restriction in most scenarios. However, an anti-TRBC2 antibody would be desirable to facilitate the analysis in a fashion similar to the staining for both kappa and lambda immunoglobulin light chains in the routine identification of clonal B cells. While an anti-TRBC2 antibody for flow cytometry is not yet commercially available, one group has reported genetically modifying the JOVI.1 clone to change its specificity from TRBC1 to TRBC2 [82], suggesting that routine assessment of TRBC2 expression might be on the horizon.

With TRBC1 added to a comprehensive T-cell panel, the diagnostic utility of each surface antigen evaluated might depend more on how clearly and frequently they separate neoplastic events from background reactive T-cells rather than on how often they are expressed in reactive versus malignant samples. For example, while CD7 and CD26 expression might not by itself be useful to distinguish reactive from malignant CD4-positive T-cells, evaluation of these antigens does result in the identification of distinct CD4-positive T-cell subsets that can be independently analyzed for clonality by TRBC1 and often lead to an optimal neoplastic cell gate. Similarly, CD5 and CD7 expression cannot by itself be used to distinguish reactive from malignant CD8-positive T-cells, but the assessment of these antigens often results in distinct subsets, which can help narrow down on the clonal population by TRBC1 analysis. Other antigens less frequently utilized in routine practice such as CCR4, PD1, CD56, CD57, CD25, and KIRDL26 might all need to be re-evaluated regarding their diagnostic utility in combination with TRBC1.

Intracellular TRBC1 staining by flow cytometry is feasible and might provide useful diagnostic information in the distinction between T lymphoblastic leukemia/lymphoma and benign thymocytes, both of which are mostly negative for surface CD3/TCR complex (manuscript in preparation). Computer-assisted data analysis could facilitate the automatic identification of clonal T-cell populations with a monophasic TRBC1 expression pattern, without the operator needing to study several gated T-cell subsets independently. Finally, many questions remain regarding the clinical significance of T-CUS, which need to be answered on large follow-up studies.

## Figures and Tables

**Table 1:** Role of T-cell receptor β chain constant region 1 (TRBC1) staining in the flow cytometric evaluation of clinical specimens.

Scenario	Utility of TRBC1 Staining
CD3^+^/TCRαβ^+^ T-cell neoplasias	Rapid demonstration of clonality on immunophenotypically distinct and expanded T-cell subsets, supporting a diagnosis of neoplasia and eliminating the need for a separate T-cell clonality assay.
Benign CD3^+^/TCRαβ^+^ T-cell subsets with immunophenotypic features concerning for neoplasia	Demonstration of TCR Cβ polytypia on benign subsets with atypical immunophenotypic features, rapidly and confidently ruling out neoplasia and preventing unnecessary additional work-up and/or misdiagnoses.
T-cell large granular lymphocytic leukemia (T-LGLL), and clonal T-cell large granular lymphocytic populations of uncertain significance (T-CUS)	Accurate identification and quantitation of clonal cytotoxic T-cell subsets, contributing to the diagnosis of T-LGLL and its distinction from T-CUS based on clone size.
Cutaneous T-cell lymphoma (CTCL)	Identification and quantitation of clonal CD4^+^ T-cell subsets, allowing for a confident diagnosis of CTCL involvement, and accurate assessment of the extent of blood involvement for staging purposes.
Fine needle aspirates and small biopsies	Rapid identification of immunophenotypically distinct T-cell clones, providing valuable information for the interpretation of limited specimens, and the need for additional work-up or an excisional biopsy.

## Data Availability

Data sharing not applicable.

## References

[1] Bjorkman P.J. (1997). MHC restriction in three dimensions: A view of T cell receptor/ligand interactions. Cell.

[2] Tonegawa S. (1983). Somatic generation of antibody diversity. Nature.

[3] Bruggemann M., White H., Gaulard P., Garcia-Sanz R., Gameiro P., Oeschger S., Jasani B., Ott M., Delsol G., Orfao A. (2007). Powerful strategy for polymerase chain reaction-based clonality assessment in T-cell malignancies Report of the BIOMED-2 Concerted Action BHM4 CT98-3936. Leukemia.

[4] Langerak A.W., Groenen P.J., Bruggemann M., Beldjord K., Bellan C., Bonello L., Boone E., Carter G.I., Catherwood M., Davi F. (2012). EuroClonality/BIOMED-2 guidelines for interpretation and reporting of Ig/TCR clonality testing in suspected lymphoproliferations. Leukemia.

[5] Wang H.W., Raffeld M. (2019). Molecular assessment of clonality in lymphoid neoplasms. Semin. Hematol..

[6] Schumacher J.A., Duncavage E.J., Mosbruger T.L., Szankasi P.M., Kelley T.W. (2014). A comparison of deep sequencing of TCRG rearrangements vs traditional capillary electrophoresis for assessment of clonality in T-Cell lymphoproliferative disorders. Am. J. Clin. Pathol..

[7] Morice W.G., Katzmann J.A., Pittelkow M.R., el-Azhary R.A., Gibson L.E., Hanson C.A. (2006). A comparison of morphologic features, flow cytometry, TCR-Vbeta analysis, and TCR-PCR in qualitative and quantitative assessment of peripheral blood involvement by Sezary syndrome. Am. J. Clin. Pathol..

[8] Morice W.G., Kimlinger T., Katzmann J.A., Lust J.A., Heimgartner P.J., Halling K.C., Hanson C.A. (2004). Flow cytometric assessment of TCR-Vbeta expression in the evaluation of peripheral blood involvement by T-cell lymphoproliferative disorders: A comparison with conventional T-cell immunophenotyping and molecular genetic techniques. Am. J. Clin. Pathol..

[9] Morice W.G., Kurtin P.J., Leibson P.J., Tefferi A., Hanson C.A. (2003). Demonstration of aberrant T-cell and natural killer-cell antigen expression in all cases of granular lymphocytic leukaemia. Br. J. Haematol..

[10] Olteanu H., Karandikar N.J., Eshoa C., Kroft S.H. (2010). Laboratory findings in CD4(+) large granular lymphocytoses. Int. J. Lab. Hematol..

[11] Wu D., Anderson M.M., Othus M., Wood B.L. (2016). Clinical Experience With Modified, Single-Tube T-Cell Receptor Vbeta Flow Cytometry Analysis for T-Cell Clonality. Am. J. Clin. Pathol..

[12] Maciocia P.M., Wawrzyniecka P.A., Philip B., Ricciardelli I., Akarca A.U., Onuoha S.C., Legut M., Cole D.K., Sewell A.K., Gritti G. (2017). Targeting the T cell receptor beta-chain constant region for immunotherapy of T cell malignancies. Nat. Med..

[13] Viney J.L., Prosser H.M., Hewitt C.R., Lamb J.R., Owen M.J. (1992). Generation of monoclonal antibodies against a human T cell receptor beta chain expressed in transgenic mice. Hybridoma.

[14] Bonilla F.A., Oettgen H.C. (2010). Adaptive immunity. J. Allergy Clin. Immunol..

[15] Davis M.M., Bjorkman P.J. (1988). T-cell antigen receptor genes and T-cell recognition. Nature.

[16] Krangel M.S. (2009). Mechanics of T cell receptor gene rearrangement. Curr. Opin Immunol..

[17] van Dongen J.J., Langerak A.W., Bruggemann M., Evans P.A., Hummel M., Lavender F.L., Delabesse E., Davi F., Schuuring E., Garcia-Sanz R. (2003). Design and standardization of PCR primers and protocols for detection of clonal immunoglobulin and T-cell receptor gene recombinations in suspect lymphoproliferations: Report of the BIOMED-2 Concerted Action BMH4-CT98-3936. Leukemia.

[18] Cushman-Vokoun A.M., Connealy S., Greiner T.C. (2010). Assay design affects the interpretation of T-cell receptor gamma gene rearrangements: Comparison of the performance of a one-tube assay with the BIOMED-2-based TCRG gene clonality assay. J. Mol. Diagn..

[19] Mahe E., Pugh T., Kamel-Reid S. (2018). T cell clonality assessment: Past, present and future. J. Clin. Pathol..

[20] Janeway C.A., Travers P., Walport M., Shlomchik M.J. (2001). The Complement System and Innate Immunity. Immunobiology: The Immune System in Health and Disease.

[21] Toor A.A., Toor A.A., Rahmani M., Manjili M.H. (2016). On the organization of human T-cell receptor loci: Log-periodic distribution of T-cell receptor gene segments. J. R Soc. Interface.

[22] Kuhns M.S., Davis M.M., Garcia K.C. (2006). Deconstructing the form and function of the TCR/CD3 complex. Immunity.

[23] Chen I.J., Chen H.L., Demetriou M. (2007). Lateral compartmentalization of T cell receptor versus CD45 by galectin-N-glycan binding and microfilaments coordinate basal and activation signaling. J. Biol. Chem.

[24] Gil D., Schamel W.W., Montoya M., Sanchez-Madrid F., Alarcon B. (2002). Recruitment of Nck by CD3 epsilon reveals a ligand-induced conformational change essential for T cell receptor signaling and synapse formation. Cell.

[25] O’Shea C.C., Thornell A.P., Rosewell I.R., Hayes B., Owen M.J. (1997). Exit of the pre-TCR from the ER/cis-Golgi is necessary for signaling differentiation, proliferation, and allelic exclusion in immature thymocytes. Immunity.

[26] Risueno R.M., Schamel W.W., Alarcon B. (2008). T cell receptor engagement triggers its CD3epsilon and CD3zeta subunits to adopt a compact, locked conformation. PLoS ONE.

[27] San Jose E., Alarcon B. (1999). Receptor engagement transiently diverts the T cell receptor heterodimer from a constitutive degradation pathway. J. Biol. Chem.

[28] Toyonaga B., Yoshikai Y., Vadasz V., Chin B., Mak T.W. (1985). Organization and sequences of the diversity, joining, and constant region genes of the human T-cell receptor beta chain. Proc. Natl. Acad. Sci. USA.

[29] Ohgami R.S., Arber D.A., Zehnder J.L., Natkunam Y., Warnke R.A. (2013). Indolent T-lymphoblastic proliferation (iT-LBP): A review of clinical and pathologic features and distinction from malignant T-lymphoblastic lymphoma. Adv. Anat. Pathol..

[30] Li S., Juco J., Mann K.P., Holden J.T. (2004). Flow cytometry in the differential diagnosis of lymphocyte-rich thymoma from precursor T-cell acute lymphoblastic leukemia/lymphoblastic lymphoma. Am. J. Clin. Pathol..

[31] Shi M., Olteanu H., Jevremovic D., He R., Viswanatha D., Corley H., Horna P. (2020). T-cell clones of uncertain significance are highly prevalent and show close resemblance to T-cell large granular lymphocytic leukemia. Implications for laboratory diagnostics. Mod. Pathol..

[32] Novikov N.D., Griffin G.K., Dudley G., Drew M., Rojas-Rudilla V., Lindeman N.I., Dorfman D.M. (2019). Utility of a Simple and Robust Flow Cytometry Assay for Rapid Clonality Testing in Mature Peripheral T-Cell Lymphomas. Am. J. Clin. Pathol..

[33] Shi M., Jevremovic D., Otteson G.E., Timm M.M., Olteanu H., Horna P. (2020). Single Antibody Detection of T-Cell Receptor alphabeta Clonality by Flow Cytometry Rapidly Identifies Mature T-Cell Neoplasms and Monotypic Small CD8-Positive Subsets of Uncertain Significance. Cytom. B Clin. Cytom..

[34] van Dongen J.J., Lhermitte L., Bottcher S., Almeida J., van der Velden V.H., Flores-Montero J., Rawstron A., Asnafi V., Lecrevisse Q., Lucio P. (2012). EuroFlow antibody panels for standardized n-dimensional flow cytometric immunophenotyping of normal, reactive and malignant leukocytes. Leukemia.

[35] Kumar B.V., Connors T.J., Farber D.L. (2018). Human T Cell Development, Localization, and Function throughout Life. Immunity.

[36] Arakawa A., Vollmer S., Tietze J., Galinski A., Heppt M.V., Burdek M., Berking C., Prinz J.C. (2019). Clonality of CD4(+) Blood T Cells Predicts Longer Survival With CTLA4 or PD-1 Checkpoint Inhibition in Advanced Melanoma. Front. Immunol..

[37] Buchholz V.R., Neuenhahn M., Busch D.H. (2011). CD8+ T cell differentiation in the aging immune system: Until the last clone standing. Curr. Opin. Immunol..

[38] Blackman M.A., Woodland D.L. (2011). The narrowing of the CD8 T cell repertoire in old age. Curr. Opin. Immunol..

[39] Khan N., Shariff N., Cobbold M., Bruton R., Ainsworth J.A., Sinclair A.J., Nayak L., Moss P.A. (2002). Cytomegalovirus seropositivity drives the CD8 T cell repertoire toward greater clonality in healthy elderly individuals. J. Immunol..

[40] Wedderburn L.R., Patel A., Varsani H., Woo P. (2001). The developing human immune system: T-cell receptor repertoire of children and young adults shows a wide discrepancy in the frequency of persistent oligoclonal T-cell expansions. Immunology.

[41] Maini M.K., Gudgeon N., Wedderburn L.R., Rickinson A.B., Beverley P.C. (2000). Clonal expansions in acute EBV infection are detectable in the CD8 and not the CD4 subset and persist with a variable CD45 phenotype. J. Immunol..

[42] Beverley P.C., Maini M.K. (2000). Differences in the regulation of CD4 and CD8 T-cell clones during immune responses. Philos. Trans. R Soc. Lond B Biol. Sci..

[43] Wack A., Cossarizza A., Heltai S., Barbieri D., D’Addato S., Fransceschi C., Dellabona P., Casorati G. (1998). Age-related modifications of the human alphabeta T cell repertoire due to different clonal expansions in the CD4+ and CD8+ subsets. Int. Immunol..

[44] Horna P., Olteanu H., Jevremovic D., Otteson G.E., Corley H., Ding W., Parikh S.A., Shah M.V., Morice W.G., Shi M. (2021). Single-Antibody Evaluation of T-Cell Receptor beta Constant Chain Monotypia by Flow Cytometry Facilitates the Diagnosis of T-Cell Large Granular Lymphocytic Leukemia. Am. J. Clin. Pathol..

[45] Garrido P., Ruiz-Cabello F., Barcena P., Sandberg Y., Canton J., Lima M., Balanzategui A., Gonzalez M., Lopez-Nevot M.A., Langerak A.W. (2007). Monoclonal TCR-Vbeta13.1+/CD4+/NKa+/CD8-/+dim T-LGL lymphocytosis: Evidence for an antigen-driven chronic T-cell stimulation origin. Blood.

[46] Rodriguez-Caballero A., Garcia-Montero A.C., Barcena P., Almeida J., Ruiz-Cabello F., Tabernero M.D., Garrido P., Munoz-Criado S., Sandberg Y., Langerak A.W. (2008). Expanded cells in monoclonal TCR-alphabeta+/CD4+/NKa+/CD8-/+dim T-LGL lymphocytosis recognize hCMV antigens. Blood.

[47] Horna P., Shi M., Jevremovic D., Craig F.E., Comfere N.I., Olteanu H. (2020). Utility of TRBC1 Expression in the Diagnosis of Peripheral Blood Involvement by Cutaneous T-Cell Lymphoma. J. Invest. Dermatol..

[48] Lamy T., Moignet A., Loughran T.P. (2017). LGL leukemia: From pathogenesis to treatment. Blood.

[49] Sanikommu S.R., Clemente M.J., Chomczynski P., Afable M.G., Jerez A., Thota S., Patel B., Hirsch C., Nazha A., Desamito J. (2018). Clinical features and treatment outcomes in large granular lymphocytic leukemia (LGLL). Leuk Lymphoma.

[50] Chan W.C., Foucar K., Morice W.G., Matutes E., Swerdlow S.H., Campo E., Lee Harris N., Jaffe E.S., Pileri S.A., Stein H., Jurgen T., Arber D.A., Hasserjian R.P., Le Beau M.M. (2017). T-cell large granular lymphocytic leukaemia. WHO Classification of Tumors of Haematopoietic and Lymphoid Tissues.

[51] Semenzato G., Zambello R., Starkebaum G., Oshimi K., Loughran T.P. (1997). The lymphoproliferative disease of granular lymphocytes: Updated criteria for diagnosis. Blood.

[52] Morice W.G., Kurtin P.J., Tefferi A., Hanson C.A. (2002). Distinct bone marrow findings in T-cell granular lymphocytic leukemia revealed by paraffin section immunoperoxidase stains for CD8, TIA-1, and granzyme B. Blood.

[53] Sandberg Y., Almeida J., Gonzalez M., Lima M., Barcena P., Szczepanski T., van Gastel-Mol E.J., Wind H., Balanzategui A., van Dongen J.J. (2006). TCRgammadelta+ large granular lymphocyte leukemias reflect the spectrum of normal antigen-selected TCRgammadelta+ T-cells. Leukemia.

[54] Neff J.L., Rangan A., Jevremovic D., Nguyen P.L., Chiu A., Go R.S., Chen D., Morice W.G., Shi M. (2018). Mixed-phenotype large granular lymphocytic leukemia: A rare subtype in the large granular lymphocytic leukemia spectrum. Hum. Pathol..

[55] Koskela H.L., Eldfors S., Ellonen P., van Adrichem A.J., Kuusanmaki H., Andersson E.I., Lagstrom S., Clemente M.J., Olson T., Jalkanen S.E. (2012). Somatic STAT3 mutations in large granular lymphocytic leukemia. N. Engl. J. Med..

[56] Jerez A., Clemente M.J., Makishima H., Koskela H., Leblanc F., Peng Ng K., Olson T., Przychodzen B., Afable M., Gomez-Segui I. (2012). STAT3 mutations unify the pathogenesis of chronic lymphoproliferative disorders of NK cells and T-cell large granular lymphocyte leukemia. Blood.

[57] Savola P., Bruck O., Olson T., Kelkka T., Kauppi M.J., Kovanen P.E., Kytola S., Sokka-Isler T., Loughran T.P., Leirisalo-Repo M. (2018). Somatic STAT3 mutations in Felty syndrome: An implication for a common pathogenesis with large granular lymphocyte leukemia. Haematologica.

[58] Jerez A., Clemente M.J., Makishima H., Rajala H., Gomez-Segui I., Olson T., McGraw K., Przychodzen B., Kulasekararaj A., Afable M. (2013). STAT3 mutations indicate the presence of subclinical T-cell clones in a subset of aplastic anemia and myelodysplastic syndrome patients. Blood.

[59] Kawakami T., Sekiguchi N., Kobayashi J., Imi T., Matsuda K., Yamane T., Nishina S., Senoo Y., Sakai H., Ito T. (2018). Frequent STAT3 mutations in CD8(+) T cells from patients with pure red cell aplasia. Blood Adv..

[60] Kucuk C., Jiang B., Hu X., Zhang W., Chan J.K., Xiao W., Lack N., Alkan C., Williams J.C., Avery K.N. (2015). Activating mutations of STAT5B and STAT3 in lymphomas derived from gammadelta-T or NK cells. Nat. Commun..

[61] Odejide O., Weigert O., Lane A.A., Toscano D., Lunning M.A., Kopp N., Kim S., van Bodegom D., Bolla S., Schatz J.H. (2014). A targeted mutational landscape of angioimmunoblastic T-cell lymphoma. Blood.

[62] Whittaker S.J., Cerroni L., Willemze R., Siebert R., Swerdlow S.H., Campo E., Harris N.L., Jaffe E.S., Pileri S.A., Stein H., Thiele J. (2017). Sezary Syndrome. WHO Classification of Tumours of Haematopoietic and Lymphoid Tissues.

[63] Cerroni L., Sander C.A., Smoller B.R., Willemze R., Siebert R., Swerdlow S.H., Campo E., Harris N.L., Jaffe E.S., Pileri S.A., Stein H., Thiele J. (2017). Mycosis Fungoides. WHO Classification of Tumours of Haematopoietic and Lymphoid Tissues.

[64] Scarisbrick J.J., Hodak E., Bagot M., Stranzenbach R., Stadler R., Ortiz-Romero P.L., Papadavid E., Evison F., Knobler R., Quaglino P. (2018). Blood classification and blood response criteria in mycosis fungoides and Sezary syndrome using flow cytometry: Recommendations from the EORTC cutaneous lymphoma task force. Eur. J. Cancer.

[65] Olsen E., Vonderheid E., Pimpinelli N., Willemze R., Kim Y., Knobler R., Zackheim H., Duvic M., Estrach T., Lamberg S. (2007). Revisions to the staging and classification of mycosis fungoides and Sezary syndrome: A proposal of the International Society for Cutaneous Lymphomas (ISCL) and the cutaneous lymphoma task force of the European Organization of Research and Treatment of Cancer (EORTC). Blood.

[66] Agar N.S., Wedgeworth E., Crichton S., Mitchell T.J., Cox M., Ferreira S., Robson A., Calonje E., Stefanato C.M., Wain E.M. (2010). Survival outcomes and prognostic factors in mycosis fungoides/Sezary syndrome: Validation of the revised International Society for Cutaneous Lymphomas/European Organisation for Research and Treatment of Cancer staging proposal. J. Clin. Oncol..

[67] Benton E.C., Crichton S., Talpur R., Agar N.S., Fields P.A., Wedgeworth E., Mitchell T.J., Cox M., Ferreira S., Liu P. (2013). A cutaneous lymphoma international prognostic index (CLIPi) for mycosis fungoides and Sezary syndrome. Eur. J. Cancer.

[68] Kelemen K., Guitart J., Kuzel T.M., Goolsby C.L., Peterson L.C. (2008). The usefulness of CD26 in flow cytometric analysis of peripheral blood in Sezary syndrome. Am. J. Clin. Pathol..

[69] Vaughan J., Harrington A.M., Hari P.N., Kroft S.H., Olteanu H. (2012). Immunophenotypic stability of Sezary cells by flow cytometry: Usefulness of flow cytometry in assessing response to and guiding alemtuzumab therapy. Am. J. Clin. Pathol..

[70] Horna P., Deaver D.M., Qin D., Moscinski L.C., Sotomayor E.M., Glass L.F., Sokol L. (2014). Quantitative flow cytometric identification of aberrant T cell clusters in erythrodermic cutaneous T cell lymphoma. Implications for staging and prognosis. J. Clin. Pathol..

[71] Haftcheshmeh S.M., Tajbakhsh A., Kazemi M., Esmaeili S.A., Mardani F., Fazeli M., Sahebkar A. (2019). The clinical importance of CD4(+) CD7(-) in human diseases. J. Cell Physiol..

[72] Pulitzer M.P., Horna P., Almeida J. (2020). Sezary syndrome and mycosis fungoides: An overview, including the role of immunophenotyping. Cytom. B Clin. Cytom..

[73] Horna P., Wang S.A., Wolniak K.L., Psarra K., Almeida J., Illingworth A.J., Johansson U., Craig F.E., Torres R. (2020). Flow cytometric evaluation of peripheral blood for suspected Sezary syndrome or mycosis fungoides: International guidelines for assay characteristics. Cytom. B Clin. Cytom..

[74] Craig F.E. (2020). It is time to adopt a multicolor immunophenotyping approach to evaluate blood for Sezary syndrome and mycosis fungoides. Cytom. B Clin. Cytom..

[75] Feng B., Jorgensen J.L., Jones D., Chen S.S., Hu Y., Medeiros L.J., Wang S.A. (2010). Flow cytometric detection of peripheral blood involvement by mycosis fungoides and Sezary syndrome using T-cell receptor Vbeta chain antibodies and its application in blood staging. Mod. Pathol..

[76] Lyapichev K.A., Bah I., Huen A., Duvic M., Routbort M.J., Wang W., Jorgensen J.L., Medeiros L.J., Vega F., Craig F.E. (2020). Determination of immunophenotypic aberrancies provides better assessment of peripheral blood involvement by mycosis fungoides/Sezary syndrome than quantification of CD26- or CD7- CD4+ T-cells. Cytom. B Clin. Cytom.

[77] Jamal S., Picker L.J., Aquino D.B., McKenna R.W., Dawson D.B., Kroft S.H. (2001). Immunophenotypic analysis of peripheral T-cell neoplasms. A multiparameter flow cytometric approach. Am. J. Clin. Pathol..

[78] Reichard K.K., Kroft S.H., Orazi A., Foucar K., Knowles D.M. (2013). Flow Cytometry in the Assessment of Hematologic Disroders. Neoplastic HematoPathol.ogy.

[79] Chen W., Kesler M.V., Karandikar N.J., McKenna R.W., Kroft S.H. (2006). Flow cytometric features of angioimmunoblastic T-cell lymphoma. Cytom. B Clin. Cytom..

[80] Jevremovic D., Olteanu H. (2019). Flow Cytometry Applications in the Diagnosis of T/NK-Cell Lymphoproliferative Disorders. Cytom. B Clin. Cytom..

[81] Berg H., Otteson G.E., Corley H., Shi M., Horna P., Jevremovic D., Olteanu H. (2020). Flow Cytometric Evaluation of TRBC1 Expression in Tissue Specimens and Body Fluids is a Novel, Sensitive and Specific Method for Assessment of T-Cell Clonality and Diagnosis of T-Cell Neoplasms. Cytom. B Clin. Cytom..

[82] Ferrari M., Baldan V., Ghongane P., Nicholson A., Bughda R., Akbar Z., Wawrzyniecka P., Maciocia P., Cordoba S., Thomas S. (2020). Abstract 2183: Targeting TRBC1 and 2 for the treatment of T cell lymphomas. Cancer Res..

